# Digital Condylar Parameter Assessment Using Cadiax^®^ 2 and Modjaw^®^

**DOI:** 10.3390/dj12110369

**Published:** 2024-11-19

**Authors:** Smaranda Buduru, Sara Hafidi, Oana Almășan, Manuela Manziuc, Manuela Tăut, Rareș Buduru, Vlad-Ionuț Nechita, Andreea Kui, Andreea Chisnoiu, Cecilia Bacali

**Affiliations:** 1 Prosthetic Dentistry and Dental Materials Department, Iuliu Hatieganu University of Medicine and Pharmacy, 400006 Cluj-Napoca, Romania; 2 Stomestet Dental Clinic, 400658 Cluj-Napoca, Romania; 3 Department of Medical Education, Medical Informatics and Biostatistics, Iuliu Hatieganu University of Medicine and Pharmacy, 400029 Cluj-Napoca, Romania

**Keywords:** sagittal condylar inclination, Bennett angle, Cadiax 2, Modjaw, TMJ digital assessment

## Abstract

**Background**: The main aim of this research was to assess the reliability of two systems designed specifically for condylar movement recording using condylar slope and Bennett angle information. The objectives were to evaluate the validity of two subsequent null hypotheses: (1) there is no significant difference between the measurements of condylar slope and Bennett angle taken at T0 (initial) and T1 (after one week) using the same equipment; (2) there is no notable difference in the values of the condylar slope and Bennett angle measurements obtained using Modjaw and Cadiax 2. **Methods**: An observational, descriptive, and prospective study was conducted with a selected group of 25 individuals (13 females and 12 males) aged between 22 and 27. **Results**: The results of Cadiax 2 and Modjaw showed excellent measurement repeatability for both parameters, with intraclass correlation coefficients (ICC) above 0.90, indicating excellent reliability between T0 and T1, both at 3 mm and 5 mm of displacement. Modjaw had an overall average value relatively higher than Cadiax 2, even though Modjaw’s condylar slope at 5mm had a significantly lower average value (37.4 ± 6.31) with an interval of 24.5–48.1, which was lower than Cadiax 2 (48.4 ± 10.6) with an interval of 30.5–68.5. Regarding the primary aim, it can be stated that both Modjaw and Cadiax 2 demonstrated excellent repeatability on their own, demonstrating robust reliability since there was no discernible difference between the T0 and T1 measurements. On the contrary, analyses of the two devices’ measured values for the secondary aim showed a considerable difference. **Conclusions**: Even though each device is reliable on its own, the absolute values that are obtained are different. Technological differences between the systems may account for these variations.

## 1. Introduction

Over the years, technological advances led to significant changes and innovations in dentistry. Innovative tools and methods that offered optimal planning and execution of dental treatments were provided. However, in the transition to new techniques and digital strategies, it has become essential that these procedures are evaluated [1,2]. Due to the large number and different techniques used, little scientific data are available to assess their precision and reliability over time. Repeatability is defined as the quality of a measurement that gives the same result if repeated under identical conditions and within a short time interval [3]. Accurate data recording and statistical validation are essential to ensure a reliable identification of medical conditions [4].

The temporomandibular joint (TMJ) comprises a very intricate structure [5]. Functional ranges of movement assessment are frequently used in clinical evaluations of mouth-opening functions [6]. Correct dental occlusion is crucial for functions such as chewing, speech, and the esthetics of the smile [7]. Therefore, any imbalance of the temporomandibular joint can lead to dental problems and functional discomfort, so its analysis is essential. Temporomandibular disorders (TMDs) are a significant cause of persistent orofacial pain, affecting an important segment of people worldwide [8,9]. The most common signs and symptoms of TMD include arthralgia, clicking or popping, limited jaw movement, and headaches [10].

Condylar parameters are of great importance in the analysis of dental occlusion and temporomandibular joint (TMJ) function [11,12]. Two of these parameters, namely condylar slope and Bennett angle, play a critical role in assessing jaw dynamics and in understanding the complex interactions between the bony and muscular components of the TMJ [11,13]. Concerning appropriate prosthodontic treatment, understanding specific condylar motions, Bennett angle, condyle inclination, and eminence orientation is crucial [14]. An essential consideration for designing prostheses involves the sagittal condylar inclination [15]. Apart from the Bennett angle and the immediate side shift, among the most significant articulator setup variables is the sagittal condylar inclination [16,17]. The condylar slope is the path of the mandibular condyle along the temporal eminentia during a protrusive movement, while the Bennett angle corresponds to the angle formed in a horizontal plane by the trajectory of the non-working (orbiting) condyle with a plane parallel to the median sagittal plane during a lateral movement on the opposite side [18].

Several techniques have been developed over the years to better appreciate the individual variations of the two posterior condylar parameters described above, like Cadiax and Modjaw, two instruments used in these assessments each based on a different principle.

There have been reports of several methods for utilizing the true horizontal plane to place the upper jaw cast to the conventional semi-adjustable articulator [19]. Identifying the sagittal and transversal condylar inclinations can be achievable using devices that monitor jaw movements [20]. Cadiax is an instrument that allows the collection of different trajectories of mandibular movements in the three anatomical planes in a computerized manner, based on electronic axiography [2]. The accuracy of the condyle movements is ensured by the verification of condylar guiding using computerized axiography devices [21,22]. Condylar movement analysis is a trustworthy method for functional evaluation that aids in the assessment of clinical conditions [19].

Various jaw-tracking technologies allow monitoring of the mandible’s static and dynamic position [23,24,25,26]. Modjaw is a tool that uses a high-frequency camera (120 Hz), which makes it possible to record and analyze occlusion in real-time, static, and dynamic settings. In fact, after acquiring the 3D models of the patient’s arches, Modjaw permits one to precisely visualize the movements of the condyles during the movements of the masticatory system [27].

This study aimed at evaluating the repeatability of two specific systems for recording condylar movements using the values of the Bennett angle and the condylar slope. Two null hypotheses were formulated, as follows: (1) there is no significant difference between the measurements of the condylar slope and the Bennett angle made at different time intervals with the same device; (2) there is no significant difference between the values of the measurements of the condylar slope and the Bennett angle made with two different devices, i.e., Modjaw and Cadiax 2.

## 2. Materials and Methods

This study was approved by the Ethics Committee of the University of Medicine and Pharmacy (number 15/29.05.2024). This study followed the Declaration of Helsinki Ethical Guidelines. Each participant signed an informed consent form. This observational, descriptive, and prospective study was conducted at the University of Medicine and Pharmacy “Iuliu Hațieganu”, Department of Prosthodontics and Dental Materials, Cluj-Napoca, Romania, during April–May 2024.

A sample of 25 subjects, including 13 females and 12 males, aged between 22 and 27 years, was randomly selected from a group of students studying dental medicine.

The inclusion criteria included subjects with intact arches; without extensive prosthetic work; who volunteered to participate in this study; knew how to perform the requested movements (propulsion and right and left laterotrusion); were not anxious or claustrophobic given the devices used for this study; and did not have diagnosed musculoskeletal problems.

The exclusion criteria included subjects with general health problems; with ongoing orthodontic or other dental treatments; with Kennedy class I, II, III, or IV edentations; who could not be manipulated in centric relation (by unimanual technique due to muscle hyperactivity), which could be verified on the screen of Modjaw and Cadiax 2; and diagnosed with temporomandibular dysfunction disorder.

**Figure 1:**
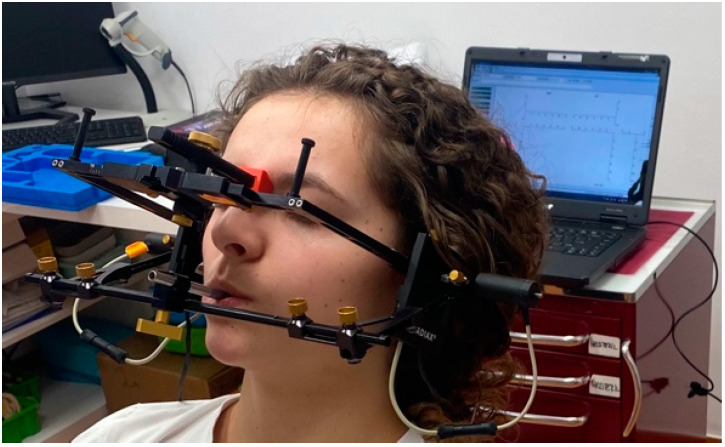
Installation of Cadiax 2.

Computerized axiography was performed by the means of Cadiax device (Figure 1) and its software program (Cadiax Compact 2 Version 2.9.2; Whip Mix Corp, Louisville, KY, USA). The Modjaw device was also used for the cinematic recording of condylar parameters.

Subjects were examined first, the T0 measurements were recorded, and the subjects were re-examined 1 week later to obtain measurements at T1. To standardize the measurements, all measurements were performed by a single operator (S.H.), and three consecutive jaw movement registrations were performed each time for both the Cadiax and Modjaw devices. One practitioner with over five years of experience (M.T.) showed the participants the expected movements and then verified and corrected each type of performed movement by the subject. Each subject exercised the movement in front of a mirror to ensure its correct understanding.

Once the movements were verified, the following movements were recorded: CR, maximum protrusion, maximum right and left laterotrusion, and maximum opening. The CR position was set as a reference position from which all the movements began and ended. The CR position was obtained by unimanual manipulation by opening and closing with an amplitude of 20 mm maximum (rotation of the condyles). To assure the same starting point, the location in CR can be visually adjusted on the screen after it has been established.

For the Cadiax 2 registration, the occlusal clutch was filled with a silicone material and fixed to the mandibular teeth (Figure 1). The patient was manipulated in CR on the clutch. The maxillary and mandibular facebows were positioned and aligned parallel to each other. The recording flags were attached, and the stylets were secured. The entire setup was connected to the Cadiax 2 system, which was linked to a computer with a dedicated software. After installation, a file was created for each participant, and a hinge axis was recorded during slight mouth movements in centric relation (CR). The participants were then instructed to perform protrusive and laterotrusive movements for further recordings, which were repeated three times.

**Figure 2:**
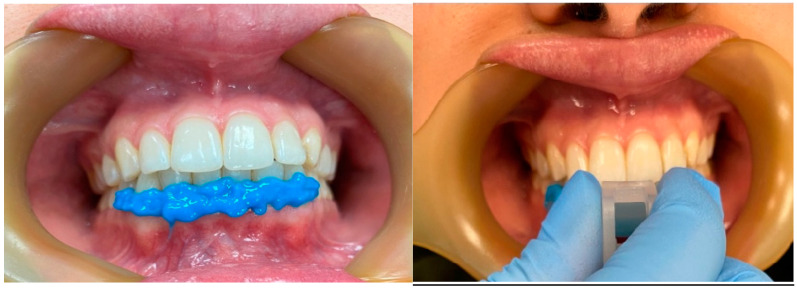
Application of the liquid dam at the tooth–gum junction and fixation of the para-occlusal fork.

Before using Modjaw, the participants’ arches and occlusion were scanned with a 3 Shape Trios 3 intraoral scanner. Para-occlusal forks were adjusted to follow the shape of the lower arch and secured using a liquid dam without interfering with occlusion. Afterwards, the butterfly reflector and pericranial arch were attached (Figure 2). The Modjaw calibration protocol consisted of four dental landmarks selected on the screen and the selection of the cutaneous points for right and left condyle and subscale points in the same patient’s mouth. Afterwards, the patient closed the mouth in maximum intercuspation, and the system validated this position. The Modjaw examination started with unimanual manipulation in the CR position. This position was validated by the software when the amplitude of each condyle was less than 1 mm^2^ on the screen and interincisal inferior point had a vertical trajectory. After validation of CR position, the participants performed the same mandibular movements as with Cadiax 2, including protrusive and right/left laterotrusive movements.

As the movements were repeated 3 times, an average was calculated each time to keep only one value. The values of the condylar slopes and the Bennett angles at 3 mm and 5 mm were collected for each device. The average values of the condylar slopes and Bennett angles at 3 and 5 mm amplitude were reported in an Excel spreadsheet.

**Figure 3:**
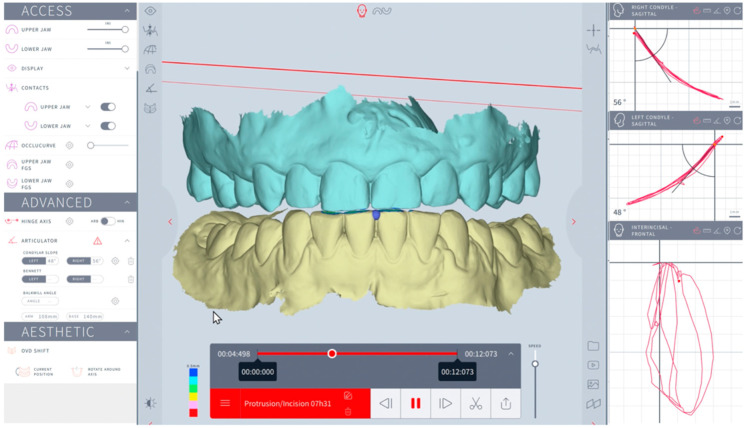
Modjaw examination: sagittal condylar inclination computation at 3 and 5 mm.

**Figure 4:**
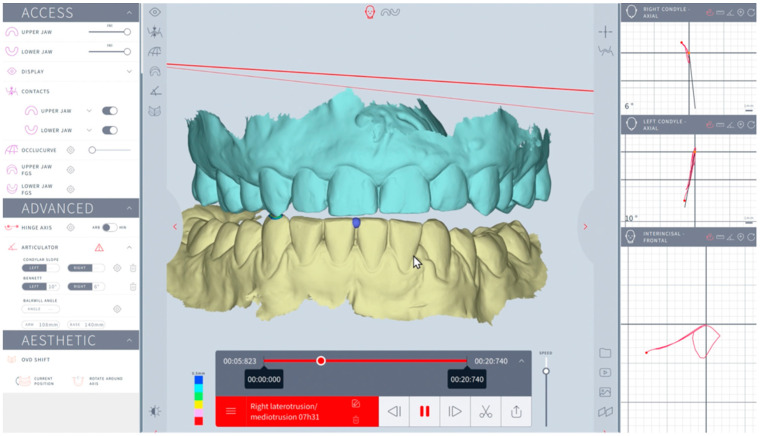
Modjaw examination: Bennett angle computation at 3 and 5 mm.

The right and left sagittal condylar inclinations were automatically calculated at 3 and 5 mm condylar displacement in the sagittal plane while three consecutive protrusive movements were shown. The right and left Bennett angles on the opposite side were automatically calculated at 3 and 5 mm condylar displacements at the axial plane after three consecutive laterotrusive motions were shown (Figure 3 and Figure 4).

### Statistical Analysis

The statistical analysis was performed using R software version 4.1.2. For the comparison of continuous variables between the groups, Student’s t-test, Wilcoxon, or Mann–Whitney U-test were used as appropriate. The data collected from the evaluations were tested for normal distribution using the evaluation of skewness, kurtosis, and the Shapiro–Wilk test for small samples. Quantitative data were presented as average values and standard deviations. Qualitative variables were presented as absolute and relative values. For the evaluation of resemblance and reliability in multiple measurements, an Intraclass Correlation Coefficient (ICC) and the associated p-value were used. The agreement between Cadiax and Modjaw measurements was evaluated through the Bland–Altman analysis. Results were considered statistically significant at *p* < 0.05.

## 3. Results

The subjects that participated in this study consisted of 52% females and 48% males.

### 3.1. Analysis of the Repeatability Between T0 and T1 of Cadiax 2 and Modjaw

#### 3.1.1. Cadiax 2

At 3 mm, the ICCs for the right and left condylar slopes were 0.974 and 0.976, respectively, while for the right and left Bennett angles, the values were 0.937 and 0.950.

At 5 mm, the ICCs for the right and left condylar slopes were 0.972 and 0.982, respectively, while for the right and left Bennett angles, the values were 0.926 for both sides (Table 1).

#### 3.1.2. Modjaw

At 3 mm, the ICCs for the right and left condylar slopes were 0.973 and 0.970, respectively, while for the right and left Bennett angles, the values were 0.954 and 0.968.

At 5 mm, the ICCs for the right and left condylar slopes were 0.946 and 0.928, respectively, while for the right and left Bennett angles, the values were 0.946 and 0.964 (Table 2).

### 3.2. Comparison Between Cadiax 2 and Modjaw

The mean values of condylar slopes and Bennett angles were analyzed with their respective standard deviations and ranges of values. At 3 mm, the values of the right and left condylar slopes were slightly higher with Modjaw compared to those with Cadiax 2. A similar trend was observed for the values of right and left Bennett angles. At 5 mm, a notable difference was observed for the left condylar slope, which was significantly lower for Modjaw in comparison to Cadiax II. For the other parameters, there was no statistically significant difference (Table 3).

**Figure 5:**
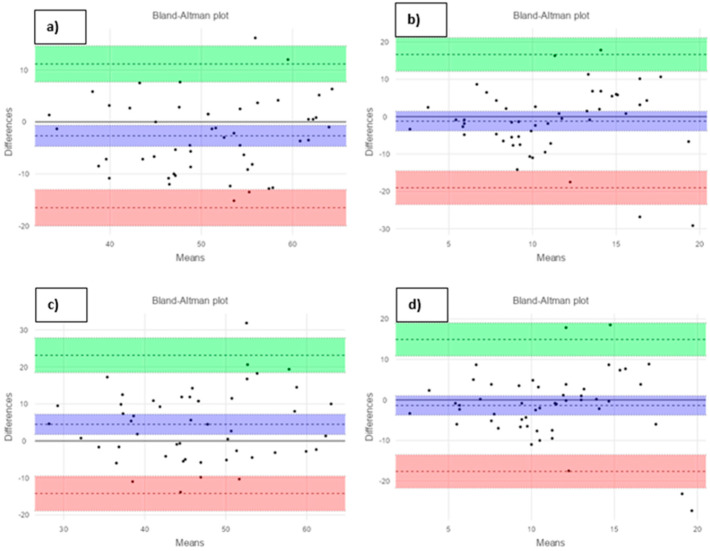
Bland–Altman graphs to evaluate the agreement between measurements with Cadiax and Modjaw: (**a**) for SCI at 3 mm; (**b**) for BA at 3 mm; (**c**) for SCI at 5 mm; and (**d**) for BA at 5 mm.

The agreement between Cadiax and Modjaw measurements was evaluated as follows: −2.67 with 95% CI (−16.5–11.15) for SCI at 3 mm; −1.19 with 95% CI (−19.01–16.63) for TCI at 3 mm; 4.47 with 95% CI (−14.2–23.16) for SCI at 5 mm; and -1.36 with 95% CI (−17.64–14.9) for TCI at 5 mm (Bland–Altman analysis, Figure 5).

## 4. Discussion

This study aimed to evaluate the repeatability of digital condylar movement measurements using two devices, Cadiax 2 and Modjaw, focusing on Bennett angles and condylar slopes.

No significant difference was observed between the initial (T0) and after-one-week (T1) measurements, both at 3 mm and 5 mm, thus confirming the first hypothesis for Cadiax 2 and Modjaw.

For the second hypothesis, statistical analyses revealed significant differences between the measurements made by the two devices, which rejects the second hypothesis.

The results of Cadiax 2 and Modjaw showed excellent repeatability of the measurements for both parameters, with intraclass correlation coefficients (ICC) greater than 0.90, indicating excellent reliability between T0 and T1, both at 3 mm and 5 mm. This result is consistent with other similar studies. In the study by Bapelle et al., the condylar slopes at 3 mm and 5 mm and the Bennett angle at 4 mm were measured by Modjaw on 22 asymptomatic subjects at 2 different sessions. Their aim was also to evaluate the repeatability of this device. The results demonstrated good to excellent repeatability of Modjaw, which supports our results [27].

As for Cadiax 2, Schiertz et al. tested the repeatability of mandibular kinematic recordings of the device by performing measurements during two separate sessions. They reported good inter-session reliability for condylar slope measurements but poor reliability for Bennett angle measurements [28].

A parallel can be drawn with the fact that for the Bennett angle the ICCs, at 3 mm as well as at 5 mm, were certainly above 0.90 but still below the ICCs of the condylar slope in our study.

In addition, Cadiax Compact uses a digital tracking method that can be influenced by positioning errors and approximations of the measured values, which would explain this variation [1].

It was also noted that the mean value of the condylar slope at 5 mm was significantly lower for Modjaw (37.4 ± 6.31) (with a range of 24.5−48.1) than for Cadiax 2 (48.4 ± 10.6) (with a range of 30.5 to 68.5). Moreover, the overall mean values of Modjaw were relatively high compared to those of Cadiax 2. This trend was demonstrated by Nigam et al., who conducted a comparative study between Modjaw and Cadiax 2 by measuring the same parameters as our study on 15 participants. Significant differences in the measurements of the condylar slope and the Bennett angle between the two devices were noted. For example, for the 3 mm condylar slope, the values measured with Modjaw were generally higher than those measured with Cadiax 2, with statistically significant differences (*p* = 0.007 for the right condylar slope and *p* = 0.010 for the left condylar slope) [29].

Previous research has demonstrated that, in comparison to Cadiax Compact 2, Modjaw measurements indicated higher transversal and sagittal condylar inclination values at 3 and 5 mm, while the analysis of the Bennett angle demonstrated an intricate association [30].

The differences in measurements can be attributed to several factors. Modjaw and Cadiax 2 methods differ not only in their tracking technology but also in their initial reference points. Cadiax 2 was used as a reference plane and Frankfurt horizontal plane, which takes the following cutaneous points into consideration: the most inferior point of the orbit and the most superior part of the tragus. In addition, the maxillary facial bow is placed in both ears, trying to approximate the center of the condyle within the device’s construction. Modjaw relies on the axio-orbital plane as a horizontal reference plane and, through its construction, defines the real hinge axis together with the anatomic centers of condyles. One possible explanation for the disparity is that the two approaches used distinctive baseline points [31]. Using different reference planes, even if between the two horizontal planes there is a small angular difference, is apparently sufficient to induce differences in the condylar measurements.

Although there were significant differences between the two devices, the question is whether these values are clinically acceptable. One study compared the accuracy and precision of Modjaw with that of an industrial scanner. It was found that their precision and accuracy were comparable [32].

As for Cadiax 2, two studies that attest that the measurement errors of Cadiax 2 are within a clinically acceptable range were found. In one of the studies, Celar and Tamaki observed differences between the measurements of Cadiax Compact and those of manually adjusted articulator devices. The measurement errors ranged from 0.4° to 2.6°, with an average of 1.2°, which remains within a clinically acceptable range for most applications [1].

In the other study directed at the evaluation of Cadiax Compact 2, a mean error of 0.44° with a maximum error of 2.5° in the left HCI setting was found, confirming sufficient accuracy for clinical use [2].

The results of the studies indicate that, although differences exist between the methods of measurement, these deviations are generally within an acceptable range for clinical use. Digital systems such as Cadiax 2 and Modjaw offer advantages in terms of reproducibility and ease of integration into dynamic digital workflows.

There are a few limitations to this research. Study population: The subjects included in this study were dental students, which facilitated the performance of the requested movements. Participants’ age range was restricted to the third life decade (22−27 years), representing a young population. All subjects were healthy and had natural teeth, without prosthetic restorations and no joint pathology. A study of a larger sample with more varied characteristics would be more representative of the general population and would allow for more precise results. Regarding the analyzed movements, this study focused mainly on the assessment of condylar slopes and Bennett angles. Moreover, other mandibular movement parameters, such as maximal laterotrusive and protrusive movements and ranges of motion, were not assessed. The analysis of these additional parameters could provide a more complete view of the performance of the devices. Duration of the study: This study was conducted over a relatively short period, which does not allow the evaluation of the long-term stability of measurements. A longitudinal evaluation would allow a better understanding of the real repeatability of these two systems over time.

Although both devices are individually reliable, there was a difference in the absolute values that were found for each of them. These variations could be explained by the technological differences between the two systems and the employed reference planes. CBCT assessment may be used to evaluate more precise values of the parameters and the acceptable variation for any recording device.

To further investigate these results, additional studies with larger and more diverse samples would be needed. The evaluation of the repeatability of the measurements over longer periods could also provide additional information on the stability of the measurements over time. Furthermore, a comparison of the performances of the two systems in real clinical conditions, including patients with various temporomandibular pathologies, could enrich the understanding of their practical applicability and the way their variations could influence the precision of the prosthetic restoration.

## 5. Conclusions

Cadiax 2 and Modjaw are two innovative devices that allow a digital and more modern analysis of mandibular kinematics. This study offers valuable knowledge on the still-underexplored repeatability and precision of these two systems. Individually, Modjaw and Cadiax 2 showed excellent repeatability with no significant difference between initial and at one-week apart measurements, confirming a high reliability. However, comparisons between the two devices revealed significant differences in the measured values.

## Figures and Tables

**Table 1:** ICCs of condylar slope means and Bennett angles with Cadiax 2.

Measurements at 3 mm	ICC (95% CI)	*p*
Right condylar slope	0.974 (0.943–0.988)	<0.001
Left condylar slope	0.976 (0.946–0.989)	<0.001
Right Bennett angle	0.937 (0.863–0.971)	<0.001
Left Bennett angle	0.950 (0.891–0.978)	<0.001
**Measurements at 5 mm**	**ICC (95% CI)**	***p***
Right condylar slope	0.972 (0.939–0.988)	<0.001
Left condylar slope	0.982 (0.959–0.992)	<0.001
Right Bennett angle	0.926 (0.842–0.967)	<0.001
Left Bennett angle	0.926 (0.841–0.966)	<0.001

CI—confidence interval.

**Table 2:** ICCs of condylar slope means and Bennett angles with Modjaw.

Measurements at 3 mm	ICC (95% CI)	*p*
Right condylar slope	0.973 (0.940–0.988)	<0.001
Left condylar slope	0.970 (0.933–0.986)	<0.001
Right Bennett angle	0.954 (0.900–0.979)	<0.001
Left Bennett angle	0.968 (0.930–0.986)	<0.001
**Measurements at 5 mm**	**ICC (95% CI)**	***p***
Right condylar slope	0.946 (0.882–0.976)	<0.001
Left condylar slope	0.928 (0.846–0.968)	<0.001
Right Bennett angle	0.946 (0.882–0.976)	<0.001
Left Bennett angle	0.964 (0.921–0.984)	<0.001

CI—confidence interval.

**Table 3:** Comparison between the mean values of condylar slopes and Bennett angles at 3 mm of Cadiax 2 and Modjaw.

Measurements at 3 mm	Average Values	*p*
Right condylar slope with Cadiax	49.7 (±8.79)	0.113 *
Right condylar slope with Modjaw	52.8 (±8.19)	
Left condylar slope with Cadiax	49.6 (±9.6)	0.034 *
Left condylar slope with Modjaw	51.9 (±8.67)	
Right Bennett angle with Cadiax	9.68 (±6.50)	0.179 *
Right Bennett angle with Modjaw	12.3 (±5.40)	
Left Bennett angle with Cadiax	11.3 (±5.75)	0.600 **
Left Bennett angle with Modjaw	11.0 (±6.59)	
**Measurements at 5 mm**	**Average values**	***p***
Right condylar slope with Cadiax	48.5 (±10.2)	0.113 *
Right condylar slope with Modjaw	50.5 (±7.21)	
Left condylar slope with Cadiax	48.4 (±10.6)	<0.001 *
Left condylar slope with Modjaw	37.4 (±6.31)	
Right Bennett angle with Cadiax	9.68 (±5.46)	0.120 *
Right Bennett angle with Modjaw	12.4 (±5.47)	
Left Bennett angle with Cadiax	10.8 (±5.41)	0.742 **
Left Bennett angle with Modjaw	10.8 (±6.32)	

(*) Student’s *t*-test. (**) Wilcoxon test.

## Data Availability

Data availability statements are available from the corresponding author upon reasonable request.
